# Catechol-O-Methyltransferase Val158Met Polymorphism on the Relationship between White Matter Hyperintensity and Cognition in Healthy People

**DOI:** 10.1371/journal.pone.0088749

**Published:** 2014-02-13

**Authors:** Mu-En Liu, Chu-Chung Huang, Albert C. Yang, Pei-Chi Tu, Heng-Liang Yeh, Chen-Jee Hong, Ying-Jay Liou, Jin-Fan Chen, Kun-Hsien Chou, Ching-Po Lin, Shih-Jen Tsai

**Affiliations:** 1 Department of Psychiatry, Taipei Veterans General Hospital, Kaohsiung, Taiwan; 2 Institute of Brain Science, National Yang-Ming University, Taipei, Taiwan; 3 Department of Biomedical Imaging and Radiological Sciences, National Yang-Ming University, Taipei, Taiwan; 4 School of Medicine, National Yang-Ming University, Taipei, Taiwan; 5 Center for Dynamical Biomarkers and Translational Medicine, National Central University, Chungli, Taiwan; 6 Department of Medical Research and Education, Taipei Veterans General Hospital, Taipei, Taiwan; 7 Health Care Group, Taipei Veterans Home, New-Taipei City, Taiwan; 8 Department of Pathology, Tao-Yuan Veterans Hospital, Tao-Yuan County, Taiwan; 9 Institute of Neuroscience, School of Life Science, National Yang-Ming University, Taipei, Taiwan; Institute of Automation, Chinese Academy of Sciences, China

## Abstract

**Background:**

White matter lesions can be easily observed on T2-weighted MR images, and are termed white matter hyperintensities (WMH). Their presence may be correlated with cognitive impairment; however, the relationship between regional WMH volume and catechol-O-methyltransferase (COMT) Val158Met polymorphism in healthy populations remains unclear.

**Methods:**

We recruited 315 ethnic Chinese adults with a mean age of 54.9±21.8 years (range: 21–89 y) to examine the genetic effect of COMT on regional WMH and the manner in which they interact to affect cognitive function in a healthy adult population. Cognitive tests, structural MRI scans, and genotyping of COMT were conducted for each participant.

**Results:**

Negative correlations between the Digit Span Forward (DSF) score and frontal WMH volumes (r = −.123, *P = *.032, uncorrected) were noted. For the genetic effect of COMT, no significant difference in cognitive performance was observed among 3 genotypic groups. However, differences in WMH volumes over the subcortical region (*P = *.016, uncorrected), whole brain (*P = *.047, uncorrected), and a trend over the frontal region (*P = *.050, uncorrected) were observed among 3 COMT genotypic groups. Met homozygotes and Met/Val heterozygotes exhibited larger WMH volumes in these brain regions than the Val homozygotes. Furthermore, a correlation between the DSF and regional WMH volume was observed only in Met homozygotes. The effect size (cohen’s f) revealed a small effect.

**Conclusions:**

The results indicate that COMT might modulate WMH volumes and the effects of WMH on cognition.

## Introduction

Cerebral white matter hyperintensities (WMH) are high-intensity areas observed on T2-weighted MR images and indicate white matter damage. Although WMH may be related to ischemia caused by chronic microvascular disease and hypoperfusion [1], [2], they commonly occur in patients with Alzheimer’s disease (AD) and mild cognitive impairment (MCI) [3], [4]. In contrast to diseased populations, most studies on non-demented elderly participants indicate that increased WMH in deep and periventricular areas may also be associated with cognitive impairment [5], [6]. A clinical-anatomic correlation study indicated that regional WMH volumes may be associated with cognitive performance using smaller regions of interest [4].

Catechol-O-methyltransferase (COMT), the postsynaptic enzyme that metabolizes released dopamine, is a critical enzyme in the metabolic degradation of dopamine in the prefrontal cortex [7]. The human COMT gene, mapped to chromosome 22q11, contains a common functional polymorphism, in which valine (Val) is substituted for methionine (Met) at the 158/108 locus on the peptide sequence [8]. The Val allele results in a substantial (4-fold) increase in enzyme activity, and may increase dopamine degradation and reduce dopamine signaling [8]. Dopamine signaling, specifically in the prefrontal cortex, is implicated in cognitive functioning. Numerous studies have demonstrated the effect of this genetic variant on neural function related to cognitive and affective processing. Several studies have shown that Met homozygous people have increased frontal cortex signal-to-noise ratios [9], [10] and improved performance in prefrontal-dependent cognitive tasks, such as working memory, whereas those with high-activity Val alleles have relatively inferior performance and inefficient dorsolateral prefrontal function [9], [10].

Egan et al [9] investigated the effect of the COMT Val158Met genotype in prefrontal-mediated cognition using the Wisconsin card sorting test (WCST) in patients with schizophrenia, their unaffected siblings, and controls. They found that participants with a low-activity Met allele had considerably fewer preservative errors on the WCST than Val-allele carriers, and that the Met allele load consistently predicted a more efficient physiological response in the prefrontal cortex. They suggested that the COMT Val allele may impair prefrontal cognition and physiology because it increases prefrontal dopamine depletion. Zinkstok et al [11] examined the relationship between COMT Val158Met polymorphism and brain anatomy in healthy young adults. They found that Met homozygotes reduced white matter density in the frontal lobe, the parahippocampal gyrus, and the corpus callosum in females, and was positively correlated with age. These results support the COMT Val158Met polymorphism effect on regulating white matter density. In addition, in a sample of mental retardation patients and healthy volunteers, Li et al [12] indicated that COMT Val158Met polymorphism may contribute to intelligence by affecting the association between cognition and the white matter architecture in the prefrontal lobe and hippocampal formation. Functional COMT polymorphism may also affect the distribution of brain white matter density and cognitive function in adults with velo-cardio-facial syndrome (VCFS) [13].

Although the severity of WMH is a crucial determinant of cognitive impairment [14] and COMT polymorphism can modulate brain morphometry, such as white matter architecture [11], [12], prior studies have not examined the effect of COMT genetic polymorphism on WMH development and modulating the relationship between WMH volumes and cognitive performance. To test the hypothesis that cognitive performance is related to regional WMH volumes and that this relationship can be modulated by COMT polymorphisms in a healthy Chinese population, we examined the correlations between regional WMHs and neurocognitive performances, evaluated the effect of the COMT genotype on regional WMHs, and determined whether the COMT genotype can modulate the relationship between regional WMHs and cognitive ability.

## Methods and Materials

### Participants

Three hundred fifteen healthy ethnic Chinese participants who satisfied the inclusion criteria were recruited from northern Taiwan (mean age: 54.9±21.8, range from 21 to 89). Any participants that met the following criteria were excluded: (1) the presence of any diagnosis on Axis I of the DSM-IV, such as mood disorders or psychotic disorders; (2) the presence of neurobiological disorders, such as dementia, head injury, stroke, or Parkinson’s disease; (3) the presence of cerebrovascular risk factors, such as hypertension, diabetes, hyperlipidemia or coronary heart disease; (4) severe medical illness, such as malignancy, heart failure, and renal failure; (5) illiteracy; (6) ferromagnetic foreign bodies or implants anywhere in the body that were electrically, agnetically, or mechanically activated.

### Clinical Assessments

All participants had sufficient visual and auditory acuity for cognitive testing after MRI scanning. The cognitive functioning of the participants was evaluated using the mini-mental state examination (MMSE) and the Wechsler Digit Span Forward (DSF) and Backward (DSB) tests. All participants had sufficient visual and auditory acuity to undergo cognitive testing. The 30-point MMSE cognitive test was designed for screening cognitive impairment in cross-cultural studies. Our research was conducted in accordance with the Declaration of Helsinki, and was approved by the Institutional Review Board of Taipei Veterans General Hospital. Written, informed consent was obtained from all the participants with an adequate understanding of the study.

### Genotyping

Genotyping of COMT Val158Met was performed using the PCR–RFLP method. In brief, a DNA fragment containing the Val/Met polymorphism in COMT was amplified by PCR with primers identical to those of Lachman et al’s report [8]. The Val/Met polymorphism was differentiated by the NlaIII restriction fragment length polymorphism analyzed on 10% polyacrylamide gel. Partial digestion and contamination amplification were ruled out by the complete digestion of an intrinsic restriction site and a blank sample in each batch of experiments, respectively.

### MRI Acquisition

All MR scanning was performed on a 3.0T Siemens MRI scanner (Siemens Magnetom Tim Trio, Erlangen, Germany) with a 12-channel head coil at National Yang-Ming University in Taiwan. High-resolution structural T1-weighted MR images (T1w) were acquired with 3D magnetization-prepared rapid gradient echo sequence (3D-MPRAGE; TR/TE = 2530/3.5 ms, TI = 1100 ms, FOV = 256 mm, flip angle = 7°, matrix size = 256 × 256, 192 sagittal slices, voxel size = 1.0 mm × 1.0 mm × 1.0 mm, no gap) for image registration, calculation of brain volumes, and brain mask generation. The T2-weighted fluid-attenuated inversion recovery (T2-FLAIR) images were acquired with multi-shot Turbo Spin Echo (TSE) sequences (2D-BLADE; TR/TE = 9000/134 ms, TI = 2500 ms, FOV = 225, flip angle = 140°, matrix size = 320×320, 42 transversal slices, slice thickness = 3 mm, ETL = 35) for WMH volume calculation. All images were acquired parallel to the anterior commissure-posterior commissure line. Each participant’s head was immobilized with cushions inside the coil to minimize motion artifacts generated during image acquisition.

### Image Analysis

To optimize the accuracy of the WMH registration procedure in voxel-wised analysis scheme, we combined the Diffeomorphic Anatomical Registration Through Exponentiated Lie Algebra (DARTEL)-based T1 VBM approach [15], [16] using Gaser’s VBM8 toolbox (http://dbm.neuro.uni-jena.de) with lesion segmentation toolbox (LST) [17] which was implemented in Statistical Parametric Mapping (SPM8, Wellcome Institute of Neurology, University College London, UK). First, all T1- and T2-weighted images were imported into the LST with default settings to generate WMH probability maps and binary maps in individual space. Second, all T1-weighted MR images were corrected for bias-field inhomogeneities, and affine registered to the tissue probability maps in the Montreal Neurological Institute (MNI) standard space (http://www.mni.mcgill.ca/) after tissue segmentation with the same generative model [18]. These affined tissue segments were iteratively registered to the group-based template, which was generated from all images included in the current study through nonlinear deformation using the DARTEL. Third, all resulting WMH maps (probability and binary) in individual space were then normalized and modulated by applying non-linear deformations to compare actual WMH volumetric measurements in MNI standard space. To investigate the relationship among genotypes of the COMT polymorphism, regional WMH, and cognitive performance, we divided the whole brain into 9 sub-regions (bilateral frontal, parietal, limbic, subcortical, temporal, occipital lobe, cerebellum, midbrain, and medulla) using WFU PickAtlas [19]. This brain atlas with 9 subregions was subsequently applied to WMH maps to localize the WMH loci in standard space automatically. Global WMH volumes and regional WMH volume in various sub-regions were subsequently extracted and calculated. To further control the effect of difference in brain size on global and regional WMH volume, total intracranial volume (TIV) for each participant was used to normalize WMH volume information into the whole brain ratio.

### Statistical Analysis

Statistical analysis was performed using the Statistical Package for Social Sciences (SPSS) software package (SPSS 18 for Windows, Chicago, IL, USA). Demographic data were compared among 3 groups (Val/Val, Val/Met, Met/Met) using a chi-squared test (for categorical variables) or analysis of variance (ANOVA) (for continuous variables) to evaluate group differences. Because TIV was associated with the amount of WMH volumes, we normalized each WMH volumes by this variable to consider the effect of brain size [4] in following statistical models. Analysis of covariance (ANCOVA) using age, sex, years of education, and TIV as covariates was subsequently analyzed to reveal the group differences in normalized WMH volumes and neuropsychological performance. To evaluate the correlation between regional WMH volume and neuropsychological performance, separate partial correlation analyses were performed in each COMT genotypic groups, controlling age, sex, and years of education as nuisance variable. We calculated the effect sizes using Cohen’s f. Bonferroni correction was used to adjust for multiple comparisons to reduce type I errors; meanwhile, uncorrected *P* values were also reported to avoid increasing type II error [20].

## Results

### Possible Correlations between Regional WMH Volumes and Cognition

The results of WMH regression analysis of 315 participants showed a negative correlation between regional WMH volumes and DSF scores in the frontal lobe (r = −.123; *P = *.032, uncorrected). The results are shown in Table 1.

**Table 1:** Correlation between cognitive performance and regional WMH volume in healthy participants.

	DSF	DSB	MMSE
Frontal Lobe	−0.123#	−0.067	−0.080
Temporal Lobe	−0.045	−0.071	−0.027
Occipital Lobe	−0.052	−0.063	−0.001
Parietal Lobe	−0.016	−0.060	−0.052
Limbic Lobe	−0.053	−0.033	−0.077
Subcortical Region	−0.069	−0.056	−0.071
Cerelebllar Region	−0.010	−0.037	0.043
Midbrain	0.076	−0.006	0.073
Medulla	−0.070	0.070	0.044
Total WMH	−0.083	−0.067	−0.070

Abbreviation: WMH: white matter hyperintensities. DSF: Digit Span Forward. DSB: Digit Span Backward. MMSE: mini-mental state examination.

Partial correlation analysis was performed controlling age, sex, years of education, and total intracranial volume as nuisance variable.

# Uncorrected *P*<.05.

### Demographics, Neuropsychological Performance, and Regional WMH Volume Among 3 COMT Genotypes

The COMT genotype distribution of 315 participants was Met/Met = 37, Val/Met = 128, and Val/Val = 150, and did not deviate from the Hardy–Weinberg equilibrium. The 3 groups did not exhibit significant differences in age, education, TIV, and all neuropsychological tests, including the MMSE, DSF, and DSB. However, a significant difference in sex was observed (*P = *.004, as shown in Table 2). Possible differences for WMH volume was observed in the subcortical region (*P* = .016, uncorrected) and whole brain (*P* = .047, uncorrected), and a trend was found in the frontal region (*P* = .050, uncorrected) among 3 COMT genotypic groups. Met homozygotes and Met/Val heterozygotes exhibited larger WMH volumes in these brain regions than the Val homozygotes (Table 3). However, none of them survive a Bonferroni correction for multiple comparison. We further evaluated the interaction between gender and COMT genotypes on WMH using two-factor ANCOVA analysis. The results showed no significant sex-by-genotype interaction effect on WMH volume (Table S1).

**Table 2:** Demographic characteristics and cognitive assessments between COMT genotypic groups in healthy participants.

	Met/Met	Met/Val	Val/Val	F or X2	P value
Demographic variables	(n = 37)	(n = 128)	(n = 150)		
Age (years)	58.8±4.12	56.4±1.86	52.5±1.75	1.74	0.177
Sex (male/female)	30/7	68/60	77/73	11.2	0.004*
Education (years)	11.3±1.07	13.0±0.51	13.3±0.47	1.86	0.157
TIV (liter)	1.39±0.01	1.36±0.01	1.38±0.13	1.93	0.147
Digit Span Forward	13.9±0.40	13.6±0.24	13.7±0.19	0.32	0.725
Digit Span Backward	6.86±0.75	7.53±0.34	8.13±0.31	1.86	0.158
MMSE	27.5±0.38	27.8±0.20	28.2±0.18	1.69	0.185

Abbreviation: TIV: total intracranial volume.

Data are expressed as Mean (SE).

*Bonferroni-corrected *P*<.05.

**Table 3:** Regional WMH volume differences between COMT genotypic groups.

	Normalized WMH Volumes			F value	P value (Uncorrected)
Anatomical Regions	Met/Met	Met/Val	Val/Val		
Frontal Lobe	0.170 (0.038)#	0.149 (0.020)#	0.092 (0.018)	3.017	0.050
Temporal Lobe	0.047 (0.013)	0.044 (0.007)	0.030 (0.006)	1.390	0.251
Occipital Lobe	0.016 (0.004)	0.013 (0.002)	0.011 (0.002)	0.834	0.435
Parietal Lobe	0.043 (0.015)	0.043 (0.008)	0.029 (0.007)	0.933	0.394
Limbic Lobe	0.031 (0.007)	0.027 (0.004)	0.016 (0.004)	2.903	0.056
Subcortical Region	0.205 (0.035)#	0.159 (0.019)#	0.106 (0.017)	4.183	0.016
Cerebellar Region	0.000 (0.001)	0.001 (0.001)	0.000 (0.001)	0.839	0.443
Midbrain	0.000 (0.000)	0.000 (0.000)	0.000 (0.000)	1.517	0.221
Medulla	0.000 (0.000)	0.000 (0.000)	0.000 (0.000)	1.384	0.252
Total WMH	0.511 (0.103)#	0.435 (0.055)#	0.289 (0.050)	3.083	0.047

Abbreviation: WMH: white matter hyperintensities.

The P values obtained by ANCOVA using age, sex, years of education, and total intracranial volume as covariates.

Data are expressed as mean (SE).

# A trend toward greater WMH volumes than the Val/Val group, uncorrected P<.05.

### Influence of COMT Val158Met on the Correlations between Regional WMH Volumes and Cognition

In the Met homozygous group, a negative correlation was observed between DSF scores and regional WMH volume in 4 subregions (frontal, limbic, subcortical, and temporal lobe) and the whole brain, and the frontal WMH volume had a higher correlation with DSF (r = 0.570, uncorrected *P* = .001, corrected *P* = .010) than other subregions (Table 4). However, the effect size revealed a small effect (Table S2). The correlation between frontal WMH volume and DSF score in three COMT genotypic groups was shown in Figure S1. No such correlation was observed in the Val homozygous and Met/Val heterozygous groups.

**Table 4:** Correlation between WMH volumes and Digit Span Forward score according to COMT Val158Met genotyping.

	Digit Span Forward					
	Met/Met		Met/Val		Val/Val	
Anatomical Regions	Correlation (r)	p-value	Correlation (r)	p-value	Correlation (r)	p-value
Frontal Lobe	−0.570	0.001*	−0.128	0.159	0.035	0.671
Temporal Lobe	−0.500	0.003*	−0.033	0.715	0.063	0.453
Occipital Lobe	−0.408	0.019	0.036	0.693	0.046	0.580
Parietal Lobe	−0.366	0.036	−0.063	0.490	0.153	0.065
Limbic Lobe	−0.502	0.003*	−0.049	0.591	0.086	0.305
Subcortical Regions	−0.508	0.003*	−0.016	0.857	0.013	0.877
Cerebellar Region	0.091	0.616	0.000	0.997	0.089	0.284
Midbrain	−0.240	0.179	0.099	0.277	0.148	0.074
Medulla	−0.056	0.758	−0.016	0.865	−0.113	0.175
Whole brain	−0.547	0.001*	−0.069	0.445	0.061	0.466

Abbreviation: WMH: white matter hyperintensities.

Partial correlation analysis was performed controlling age, sex, years of education, and total intracranial volume as nuisance variable. All of the p-values showed in this table were unadjusted p-values.

*Bonferroni-corrected *P*<.05.

## Discussion

This is the first study to examine the effect of the COMT gene on the relationship between regional WMH volume and cognitive performance. The results indicate a negative correlation between frontal WMH and cognition, and that the COMT gene can modify WMH development and the relationship between WMH volume and cognition. Compared with Val homozygotes, the Met/Met homozygotes and Met/Val heterozygotes had a larger WMH volume at several brain regions, including the frontal region, subcortical region, and the whole brain. Although no significant difference in WMH volumes was observed between Met homozygotes, Met/Val heterozygotes, and Val homozygotes after correction for multiple testing, a trend toward a dose-dependent effect of the Met allele on WMH volumes was observed, and Met homozygotes exhibited larger WMH volumes than the other 2 genotypes. Finally, a negative correlation between the frontal WMH volume and cognition was observed in Met/Met homozygotes, but not in Val homozygotes or Met/Val heterozygotes. Moreover, the WMH volumes over other 3 subregions (limbic, subcortical, and temporal lobe) and the whole brain were also correlated with DSF performance in Met homozygotes, and the frontal WMH volume exhibited higher correlation with DSF than other subregions.

A significantly negative correlation between regional WMH volumes and DSF scores was observed in the frontal lobe. Schmithorst et al [21] found a positive correlation between cognition and the white matter architecture in several regions of the frontal lobe in a healthy pediatric population. In middle-aged and elderly people, frontal white matter lesions (WMLs) were significantly associated with cognitive impairment [22], [23]. The role of the frontal lobe in higher cognitive functions, such as working memory, attention control, reasoning, and temporal ordering of spatial and nonspatial events, has been extensively examined in previous fMRI and PET studies, and activation of the frontal lobe with a few of these cognitive tasks was related to cognitive performance [24], [25]. Moreover, involvement in the DSF task activated several areas in the frontal cortex in functional studies [26]. These results may explain the correlation between frontal WMH volumes and DSF scores observed in this study. DSF was more sensitive to the presence of WMH than other neuropsychological measurements. Shin et al [27] obtained similar results; that is, DSF performance was significantly correlated with the burden of cholinergic WMH in patients with Parkinson’s disease. DSF may serve as a valuable early-warning screening tool in community and health care settings because of the apparent sensitivity of DSF to normal cognitive aging [28] and MCI [29], and evidence that this measure predicts conversion to dementia over several years [30]. Regional WMLs associated with deficits in other tests (DSB, MMSE) were not identified; however, these deficits may not be directly related to WMLs, but rather to cortical atrophy. For example, DSB is a more sensitive neuropsychological test than DSF in detecting cortical thinning in patients with MCI and AD [31].

Met/Met homozygotes and Met/Val heterozygotes had more WMHs than Val homozygotes in the frontal region, subcortical region, and the whole brain. Although no statistically significant difference in WMH volumes was observed between Met homozygotes and Met/Val heterozygotes, a dose-dependent effect of the Met allele on WMH volume was observed, and Met homozygotes exhibited larger WMH volumes than the other genotypes. Prior studies have examined the relationship between the COMT genotype and white matter architecture in children and adolescents [32] and healthy adults [33]; their results are similar to those obtained in this study. Thomason et al [32] found that the Met allele was associated with the disruption of white matter architecture at the frontal cortex. Liu et al [33] examined other haplotypes in the COMT gene that cover a wider range of protein expression, and found that the groups with lower enzymatic activity (ie, higher dopamine levels) had inferior white matter integrity in the bilateral prefrontal lobes. The results may be attributed to the following factors. First, the increased dopamine activity in the frontal cortex may cause oligodendrocyte damage, demyelination, and WML formation [34], [35]. In an animal study, a high level of dopamine in the frontal cortex caused by cuprizone, a copper chelator, preceded white matter damage in the same brain region, and this damage was prevented by anti-dopaminergic agents [34], [35]. In addition, amphetamine abusers had a greater prevalence of WMH than control participants, as well as greater severity of periventricular and deep WMH, primarily in the frontal lobe [36]. Amphetamine-induced dopamine releases lead to accumulation of reactive oxygen species and severe oxidative stress, which may cause white matter damage [36]. Second, the COMT Met allele may be associated with subtle physiological changes over time, such as mean arterial pressure [37], and these changes may be vital for brain hypoperfusion and WMH formation [38]. Higher dopamine levels lead to perfusion abnormalities in several brain regions, such as the frontal and subcortical areas, resulting in decreased oxygen delivery [39], which may induce oligodendroglial cell damage and WMH formation [40]. In addition to conventional cerebrovascular risk factors, potential risk factors for the development of WMLs include increased plasma homocysteine, decreased serum tryptophan, hyperinsulinemia, and hyperfibrinogenemia [38]. These factors may be highly associated with brain dopaminergic systems [41], [42]. Despite we excluded participants with conventional cerebrovascular risk factors, such as hypertension, diabetes, hyperlipidemia, and coronary heart disease, COMT Val158Met polymorphism may modulate WMH volume thought its’ impact on subtle physical changes or potential risk factors that were not considered in current study. Because the Met allele has an additive effect on COMT protein abundance and enzyme activity in the frontal cortex [8], [43], the results regarding a trend toward a dose-dependent effect of the Met allele on WMH volume may be reasonable. Third, in addition to the frontal lobe, significant interconnections between the prefrontal dopaminergic fibers and subcortical regions have been implicated in white matter development in adolescents [44], and the frontal-subcortical circuit with dopaminergic innervation may contribute to the formation of WMLs during cerebral aging [38], [45]. Because the frontal dopaminergic fibers may have extensive outgoing connections to several brain regions [45], COMT polymorphism may modulate subcortical and global WMH volumes through its effect on frontal dopaminergic neurotransmission. Therefore, Met carriers, especially Met homozygotes, with higher levels of brain dopamine may result in greater WMH volumes in several brain regions.

Another major finding of this study was that a negative correlation between the DSF and frontal WMH volume was observed only in Met homozygotes (r = 0.570, P = .001, corrected *P* = .010). Except for the frontal lobe, WMH volumes in other 3 subregions and the whole brain were correlated with DSF performance in Met homozygotes, and the frontal WMH volume showed higher correlation with DSF than other brain subregions (Table 4). Several studies have demonstrated that increased dopamine activity in the frontal cortex may cause WML formation, which can disrupt cognitive performance [46], [47]. Homozygosity for the low-activity (Met) allele leads to a 3- to 4-fold reduction in enzymatic activity compared with the high-activity (Val) allele. Therefore, the Met homozygotes may have excessive dopamine above the optimal range, resulting in greater WMH volumes, and are more vulnerable to the WMH burden on cognition. This may explain why the correlation between DSF and the frontal WMH volume was observed only in Met homozygotes.

In Met homozygotes, the correlation between DSF and regional WMH volume was observed in the frontal region and other 3 brain regions (limbic, subcortical, and temporal lobe). The frontal dopaminergic fibers may have extensive outgoing connections to several brain regions, and dopaminergic neurotransmitters and receptors are widely distributed and expressed throughout the brain [45]. COMT protein and enzyme activity exhibited widespread expression in mammalian brains [48], [49]. Since increased dopamine levels are associated with a loss of dopamine transporters, dopamine receptors, and dopamine synthesis [50], [51], and such changes in the dopaminergic system over the whole brain are also involved in the aging process and cognitive deficit [52], [53], it is not surprising that a correlation between WMH volume and DSF performance was observed over all brain subregions in the Met homozygotes. Moreover, the rate of age-related changes in the brain dopaminergic system is considerably faster in the frontal cortex compared to other brain regions [52], and cognitive aging in healthy people is accompanied by WML development, which first occurs in the frontal cortex [54]. The frontal WMH volume has a stronger correlation with DSF than the other brain regions. These results reinforce the assumption that the frontal lobe may be more vulnerable to the effects of WMLs on cognition than other brain regions, and this may be partially attributed to dopaminergic neurotoxicity regulated by the COMT genotype.

Despite COMT Val158Met polymorphism affected the correlation between DSF and regional WMH volumes, no significant effects of this polymorphism on cognitive performance were observed. This phenomenon was observed in prior studies [12]. In the genetic study of complex cognitive problems, the structural and functional features of the brain are considered intermediate phenotypes or endophenotypes, and may be more sensitive to the effect of a genotype than performance at the behavioral level, such as in cognitive tests. This difference facilitates determining the role of gene polymorphisms, such as COMT Val158Met, in the brain basis for cognition (eg, white matter structure) than in the cognition itself.

This study included a relatively large sample, a homogenous population (ethnic Chinese), MRI ratings performed in a single center, and regional information on WMH. Sufficient sample sizes are required for genetic imaging studies; the sample size used in this study met the requirements recommended by previous researchers [55]. This study was limited by several factors. First, the cross-sectional design caused difficulty in determining whether WMH leads to a cognitive deficit or other insult leads to a change in WMH and cognition simultaneously. Future studies must address this issue. Second, we excluded participants with cerebrovascular risk factors, such as hypertension, diabetes, hyperlipidemia, and coronary heart disease, which can influence hyperintensity progression. However, we did not capture other potential contributing risk factors, such as cigarette use. These factors may have affected the results. Third, we examined only COMT Val158Met polymorphism. Several other COMT SNPs (eg, rs4633, rs737865, and rs165599) can also affect gene expression, and haplotypes comprising these SNPs may have a more reliable effect on COMT gene expression than only Val158Met [56], [57]. Further studies are required to classify participants according to COMT haplotypes and explore their role in the association between WMH and cognitive ability. Fourth, COMT Val158Met polymorphism may be in linkage disequilibrium with the associated allele instead of having a direct effect on the WMH volume. This type of linkage may vary among differing populations, and can confound the generalization of findings based on a homogenous ethnic Chinese cohort, such as that used in this study. Fifth, due to the small effect size in current study, it is more difficult to distinguish between a real effect of COMT Val158Met polymorphism and random variation. Finally, we performed multiple tests in detecting the difference of WMH between groups in several regions simultaneously, but failed to meet the criteria of Bonferroni correction, and did not exclude the possibility of false positive results. Independent studies are required to further validate the findings.

In conclusion, this study found a negative correlation between frontal WMH volumes and cognitive performance in Met homozygotes. Moreover, COMT Val158Met polymorphism might modulate the WMH volume and vulnerability to the regional WMH burden on cognition. These results further suggest that regional WMH may be valuable imaging endophenotypes for genetic studies on cognitive ability.

## Supporting Information

Figure S1 **The correlation between frontal WMH volume and Digit Span Forward score in COMT genotypic groups.**
(TIF)

Table S1 **COMT genotype-by-sex interaction effect on regional WMH volume.**
(DOCX)

Table S2 **Effect size of each dependent variable.**
(DOCX)
