# Supplementary figures and images for: Catechol-O-Methyltransferase Val158Met Polymorphism on the Relationship between White Matter Hyperintensity and Cognition in Healthy People
Source: PLoS One. 2014 Feb 13;9(2):e88749. doi: 10.1371/journal.pone.0088749 (PMC3923794; doi:10.1371/journal.pone.0088749)

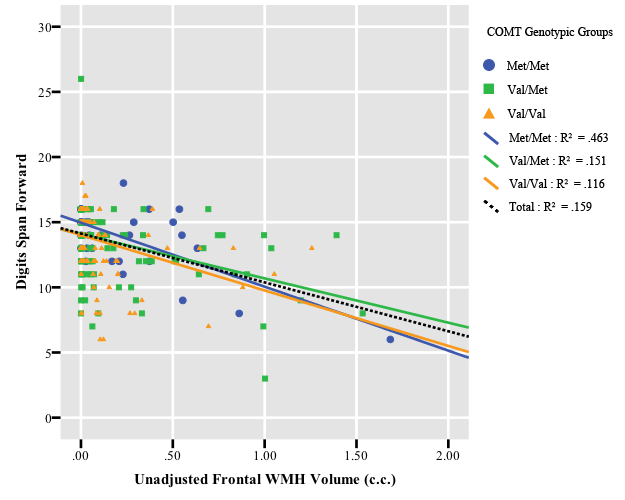

Supplement: Figure S1 — The correlation between frontal WMH volume and Digit Span Forward score in COMT genotypic groups. (TIF) [file pone.0088749.s001.tif]
