# Supplementary material for: Catechol-O-Methyltransferase Val158Met Polymorphism on the Relationship between White Matter Hyperintensity and Cognition in Healthy People
Source: PLoS One. 2014 Feb 13;9(2):e88749. doi: 10.1371/journal.pone.0088749 (PMC3923794; doi:10.1371/journal.pone.0088749)
Supplement: Table S1 — COMT genotype-by-sex interaction effect on regional WMH volume. (DOCX) [file pone.0088749.s002.docx]

Table S1. COMT genotype-by-sex interaction effect on regional WMH volume.

| WMH in Specific Anatomical Regions | Genotype-by-Sex Interaction | |
| --- | --- | --- |
|  | F-value | P-value |
| Frontal Lobe | 0.527 | 0.591 |
| Temporal Lobe | 0.481 | 0.427 |
| Occipital Lobe | 0.232 | 0.793 |
| Parietal Lobe | 0.436 | 0.647 |
| Limbic Lobe | 0.854 | 0.335 |
| Subcortical Region | 1.096 | 0.618 |
| Cerebellar Region | 0.650 | 0.523 |
| Midbrain | 0.419 | 0.658 |
| Medulla | 0.670 | 0.513 |
| Global WMH | 0.757 | 0.470 |

Abbreviations: WMH= White matter hyperintensities. WMH volumes used in each GLM were already normalized by total intracranial volume (TIV) to account for the different brain sizes.
