# Supplementary material for: Catechol-O-Methyltransferase Val158Met Polymorphism on the Relationship between White Matter Hyperintensity and Cognition in Healthy People
Source: PLoS One. 2014 Feb 13;9(2):e88749. doi: 10.1371/journal.pone.0088749 (PMC3923794; doi:10.1371/journal.pone.0088749)
Supplement: Table S2 — Effect size of each dependent variable. (DOCX) [file pone.0088749.s003.docx]

**Table S2.** Effect size of each dependent variable

| **Dependent Variables** | **Partial Eta Squared** | **Cohen’s f** |
| --- | --- | --- |
| Frontal Lobe | 0.020 | 0.143 |
| Temporal Lobe | 0.010 | 0.101 |
| Occipital Lobe | 0.004 | 0.063 |
| Parietal Lobe | 0.009 | 0.095 |
| Limbic Lobe | 0.020 | 0.143 |
| Subcortical Region | 0.026 | 0.163 |
| Cerebellar Region | 0.004 | 0.143 |
| Midbrain | 0.008 | 0.063 |
| Medulla | 0.008 | 0.090 |
| Total WMH | 0.020 | 0.090 |
| DSF | 0.009 | 0.095 |
| DSB | 0.002 | 0.045 |
| MMSE | 0.004 | 0.063 |
